# Predictors of Literacy and Attitudes Toward Reading Among Syrian Refugee Children in Jordan

**DOI:** 10.1007/s13158-022-00334-x

**Published:** 2022-09-09

**Authors:** Kristin Hadfield, Mays Al-Hamad, Rinad Bakhti, Rana Dajani, Amal El Kharouf, Julia Michalek, Joana Mukunzi, Lina Qtaishat, Tanvi Sethi, Sophie von Stumm, Isabelle Mareschal

**Affiliations:** 1 School of Psychology, Trinity College Dublin, Dublin, Ireland; 2 Taghyeer, Amman, Jordan; 3 Department of Biological and Experimental Psychology, Queen Mary University of London, London, UK; 4 Department of Biology and Biotechnology, Faculty of Science, The Hashemite University, Zarqa, Jordan; 5 Centre for Women Studies, University of Jordan, Amman, Jordan; 6 Vulnerability, Trauma, Resilience and Culture Lab, University of Ottawa, Ottawa, Canada; 7 Department of Education, University of Oxford, Oxford , UK; 8 Department of Education, University of York, York, UK

**Keywords:** Syrian refugee, Literacy, Schooling, Jordan, Reading attitudes

## Abstract

Refugee children often face disruptions to their education before and during displacement. However, little is known about either levels or predictors of refugee children’s literacy or about their attitudes toward reading in low- or middle-income countries. To address this, we conducted in-home literacy assessments using the Holistic Assessment of Learning and Development Outcomes with 322 Syrian refugee mother–child dyads who lived in Jordan (child age range 4–8 years, *M* = 6.32 years, 50% female). Overall, the children had quite low levels of literacy, although they indicated a strong enthusiasm for reading. Child age, maternal education, and maternal ability to read all predicted child literacy, although maternal literacy predicted it only among children enrolled in school. Among those enrolled in school (64.9% of the total sample, 88.7% of those aged ≥ 6), students attending hybrid classes had better literacy than those attending either solely in-person or solely online, although the frequency of school attendance did not predict literacy. A less consistent pattern emerged for predicting children’s attitudes toward reading. Our results suggest an urgent need to improve literacy skills among refugee children in Jordan, as well as a need for validated measures of attitudes toward reading for use with Arabic-speaking youth.

**Supplementary Information:**

The online version contains supplementary material available at 10.1007/s13158-022-00334-x.

Over 1% of the world's population is forcibly displaced, and more than half of those displaced are children under the age of 18 (UNHCR, 2021). Most of these children live in low- or middle-income countries next to the country from which they or their family fled (UNHCR, 2021). The timely realization of developmental milestones is often disrupted for these children, who face major adversities at multiple levels. In this paper, we focus on child literacy and attitudes toward reading in a group of Syrian refugees in Jordan.

The Syrian refugee crisis is the largest refugee crisis since World War II, with more than 6.7 million refugees forced to flee Syria since 2011 (UNHCR, 2021). Currently, there are 673,188 Syrians taking refuge in Jordan, of whom more than 80% live outside of demarcated camps (UNHCR, 2022). Syrian refugees in Jordan face substantial challenges: 85% live below the poverty line and 94% of those under the age of five are multi-dimensionally poor, meaning that they are deprived of basic needs like education, health, water and sanitation, and child protection and safety (UNICEF, 2018). Double-shifted schools are common: 69% of the Syrian refugee children in Jordan attend these double-shifted schools, with Jordanian children attending in the morning and Syrians attending in the afternoon (Ministry of Education, 2018). While increasing access to schooling for all, double shifting introduces many challenges. These include less time in formal educational settings for both refugee and non-refugee children, reduced ability for refugees and non-refugees to mix and make social connections, and fewer educational resources in school for the Syrians (Cochran, 2020; Salem, 2021).

The overall literacy rate for adolescents in Jordan is high (> 99% among those aged 15–24, World Bank, 2021), as was the rate in Syria prior to the war (¬92% among those aged 15–24, UNESCO, 2021). However, less is known about levels of literacy among pre-adolescent children in Jordan or, indeed, anywhere the Middle East and North Africa region—particularly among those who are forcibly displaced. Extant evidence from Lebanon suggests that literacy rates are quite low among Syrian refugee children, with a large proportion of 3–15-year olds unable to identify any letters or words in a direct literacy assessment (Krupar, 2019). There are challenges for refugee and non-refugee literacy education acrossq the Middle East and North Africa region and internationally; of particular importance may be limits on access to primary and secondary education for refugee children and adults (Eghbaria-Ghanamah et al., 2020; Saiegh-Haddad & Everett, 2017; Sultana, 2007; UNHCR, 2016; Wofford & Tibi, 2018). Given double-shifted schools and other challenges to Syrian refugee children in Jordan’s ability to access high-quality education (Dhingra, 2019), these children are at risk for poor literacy, which exacerbates disadvantage: Good literacy is key for children to acquire information and knowledge and to develop cognitive competencies. It is critical to assess literacy among Syrian refugee children in Jordan to know whether literacy interventions should be implemented.

While we know little about literacy among Syrian refugee children, we know even less about their attitudes toward reading. We did not find any papers which examined child attitudes toward reading among Syrian refugees, in Jordan or otherwise. This is problematic because attitude toward reading is a key affective factor predicting literacy, reading skills, and academic success throughout the lifespan (Davis et al., 2018; Nootens et al., 2019; Schiefele et al., 2012). Without understanding levels or predictors of reading motivations or attitudes toward reading in this population, it is difficult to implement effective interventions.

Optimum classroom environment, peer-to-peer learning, developmentally appropriate learning materials, and positive teacher–student relationships are enablers of children’s formal learning and educational development (United Nations, 2021). When schooling is disrupted, the influence of children’s home environments increases for their educational development. Children whose parents and caregivers have less education and low or no literacy often have lower levels of literacy themselves (Menheere & Hooge, 2010; Save the Children, 2016). Stimulating and supportive learning environments support language development, while also allowing for healthy self-regulatory practices and motivation required for school readiness and success (Gottfried, 2013). Even before COVID, Syrian refugee girls in the Middle East were less likely to attend primary or secondary school (Charles & Denman, 2013; Sirin & Rogers-Sirin, 2015, although this pattern was more varied in Jordan, see Human Rights Watch, 2020 and UNICEF, 2020). Many Syrian refugee households in Jordan have limited or no access to computers or the internet (Caswell, 2019; Michalek et al., 2022; Panter-Brick et al., 2021) —or where they have this access, it may be limited to one smartphone for the household—which is a hurdle for children’s involvement in online schooling (El-Abed & Shabaitah, 2020). In these cases, parents had to choose when each of their children would be able to engage with their online classes; it is possible that these decisions may be underpinned by gender-based educational and employment expectations for the future (Charles & Denman, 2013; El-Abed & Shabaitah, 2020; Krafft, Assaad, & Pastoor, 2021). This previous work suggests that literacy-promoting resources and processes at the child-, parent-, and household-level may differ between Syrian refugee boys and girls.

## The Current Study

To address these gaps in knowledge, in this paper, we answer the following research questions: *First,* what are the levels of literacy among 4–8-year-old Syrian refugee children in Jordan? *Second,* which child-, mother-, household-, and school-level factors are associated with literacy and attitudes toward reading among these children? *Finally*, does the association of these child-, mother-, household-, and school-level factors with literacy and attitudes toward reading differ by the child’s sex?

## Method

### Participants

We sampled 322 Syrian refugee mother–child dyads living in Amman (*n* = 236 dyads) and Zaatari (*n* = 86 dyads) as part of a larger wait-list cluster randomized controlled trial. Participants were sampled through five local community-based organizations in Amman and through two We Love Reading (WLR) reading ambassadors in Zaatari. To be included in the study, ‘mothers’ had to be a Syrian refugee who was the primary female caregiver for at least one child between the age of 4 and 8 years old. In practice, all but one were the study child’s biological mother, with the other a paternal grandmother taking part in the study as the child’s biological mother was deceased. We refer to these female caregivers as mothers throughout the paper. Where a mother had more than one child in this age range, the child closest to 6 years old was chosen as the study child. Although we set this limitation, upon collecting data from families—including their official documents—we discovered that a small number of the children were 3 (*n* = 2) or 9 years old (*n* = 1); these participants were retained for analysis. For more information on the randomized controlled trial, see the study pre-registration (Hadfield & Mareschal, 2020) and for study materials, see the project OSF page at https://osf.io/gcv5z/.

Mothers ranged in age from 20 to 55 years old (*M* = 32.61, *SD* = 7.02). The children were 6.32 years old on average (*SD* = 1.18) with an equal gender split (50.0% female). The mothers had 4.24 children on average (*SD* = 1.93). All mothers spoke Arabic fluently, and it was the first language for all study children. Most mothers had completed between 7 and 12 years of schooling (53.1%), with smaller proportions having no formal education (10.0%), between 1 and 6 years of schooling (27.2%), and at least some tertiary education (9.7%). Few mothers were employed (10.9%); of those that were, there was an approximately equal split between full-time (48.6%) and part-time (51.4%) employment.

Most of the families had been in Jordan for 8 or 9 years at the time of data collection, corresponding with the largest waves of displacement from Syria between 2012 and 2014. This means that most children in this study were born in Jordan. Participating families lived in difficult circumstances, with household sizes ranging from 3–15 people (*M* = 6.77, *SD* = 2.22), up to 4 families per house (*M* = 1.23, *SD* = 0.52), and an average of just 2.71 rooms (*SD* = 0.77, aside from the kitchen or bathroom) per household. The study child’s father lived in the home with the child in 89.1% of the families; where not in the home this was because of divorce (4.6% of dyads), the father had passed away (1.3%), the father was missing (0.8%), or another reason.

### Procedures

The research team in Jordan reached out to community-based organizations in different neighborhoods of Amman and to two women who had previously worked with the NGO WLR in Zaatari camp; these organizations and women were asked for lists of Syrian refugee women who used their services who might meet the eligibility criteria and to hold events at their organizations to sign up potential participants. From these lists, mothers were contacted by phone to see if they would be interested in taking part. Whichever 4–8-year-old child they had closest in age to 6 years old was sampled for this study.

This research received ethical approval from [blinded] in January 2021 (01E/2020/10). Mothers were informed about the study through community-based organizations, were sent information sheets, and then a data collection appointment was arranged if they agreed to take part. Two fieldworkers visited each household, with one collecting data from the mother and the other simultaneously collecting data from the child. Before data collection began, mothers consented to their and their child’s participation, and the child provided assent. Fieldworkers then collected data orally in participants’ homes, inputting the data directly into KoBoToolbox.

All fieldworkers were female to avoid participants’ potential concerns with unrelated men visiting their household, in line with other work in this region (e.g., Al-Makhamreh & Lewando-Hundt, 2008; Panter-Brick et al., 2020). Fieldworkers were native Arabic speakers who had conducted research in Jordan before. They were trained for one week before data collection, practiced data collection processes with each other, and then conducted pilot data collection with 10 families in January 2021, before commencing the full data collection between January and May 2021.

### Measures

All measures were asked orally of participants in Arabic. Where possible, we used measures which had been developed, validated for, or used with Arabic-speaking refugees in the Middle East and North Africa region. Where this was not possible, items and response options were translated to Arabic by author LQ, back translated by the field officer, and then there was a discussion between the translators and other research team members to determine the final wording. In some cases, this involved minor changes to the content of questions, to keep the content understandable and appropriate for this context.

#### Demographics

Mothers were asked to indicate their age, education level, and first language. They were also asked to self-report on their literacy with the dichotomous question, “Do you know how to read?” (No = 0, Yes = 1). They were asked their child’s age, sex, and first language.

#### Schooling

Mothers reported on the child’s schooling. They were asked if the child had ever been in school, if the child was currently enrolled in school, what grade the child was enrolled in at the time of data collection, if the child “went to school in person, online, or both ways,” and how often the child attended classes (never [1], rarely [2], sometimes [3], often [4], always [5]). Where the child attended class in person, the mothers were not asked how often they attended classes because school attendance is mandatory, and so we have coded the children attending school in-person as “always” for the class attendance item.

#### Reading

To assess exposure to reading in the home, children were asked “In the last week, have you seen someone from your family members reading at home?”. Response options were “No” (0) or “Yes” (1).

#### Poverty

The 12-item Household Wealth Index is a checklist measure developed to measure relative poverty in the region (Panter-Brick et al., 2009, 2018). Mothers were asked to report if their household had each of the following: TV, satellite, smartphone, car, refrigerator, computer, oven with gas, bedframe (not only a mattress), washing machine, heater, fan, and water heater. Possible scores could range from 0–12, with actual responses ranging from 3–12.

##### COVID

Data were collected between January and May 2021, during which time there were different levels of COVID restrictions in Jordan. The restrictions placed by the government on movement and school openings were recorded by LQ. Where there was a curfew in place which limited movement at the time of data collection, this was scored as 1; where there were no curfews, this was scored as 0.

#### Child Love of Reading

Children’s attitudes toward reading were assessed through both mother- and child-report. Mothers completed the 9-item ‘parents’ perception of their child’s attitude toward reading’ (PPCATR) subscale of the Parents Digital Literacy Questionnaire, developed in Turkey by Ozturk and Ohi (2018). Items include “My child shows emotional reactions when I read to him or her” and “My child wants to be read to,” with a 5-point Likert scale ranging from “Never” (0) to “Always” (4). The PPCATR had acceptable internal reliability in our sample (α = 0.722).

Children completed two measures about their attitudes toward reading: the 4-item efficacy for reading and 3-item reading orientation subscales of the Young Reader Motivation Questionnaire (YRMQ, Coddington & Guthrie, 2009) and the 8-item attitude subscale of the Reader Self-Concept Scale (RSCS, Chapman & Tunmer, 1995). The YRMQ was developed in English in the United States and consists of items such as “Are you good at remembering words?” and “Is it fun for you to read books?”, with Likert options of “No, never” (1), “No, not usually” (2), “Yes, usually” (3), and “Yes, always” (4) (potential range 7–28). Because our participants ranged in age from 4–8 years old and we were not sure of their familiarity with Likert measures, we chose to also use the RSCS which has dichotomous (0 = no, 1 = yes) response options (potential range 0–7). The RSCS was developed in English in New Zealand and includes items such as “Do you feel good when you read?” and “Do you like reading to yourself?”. Both the YRMQ and RSCS had good internal reliability (α = 0.795 and 0.781, respectively). Higher scores on all attitude toward reading measures indicate that the child had a more positive attitude toward reading.

#### Child Literacy

**Fig. 1:**
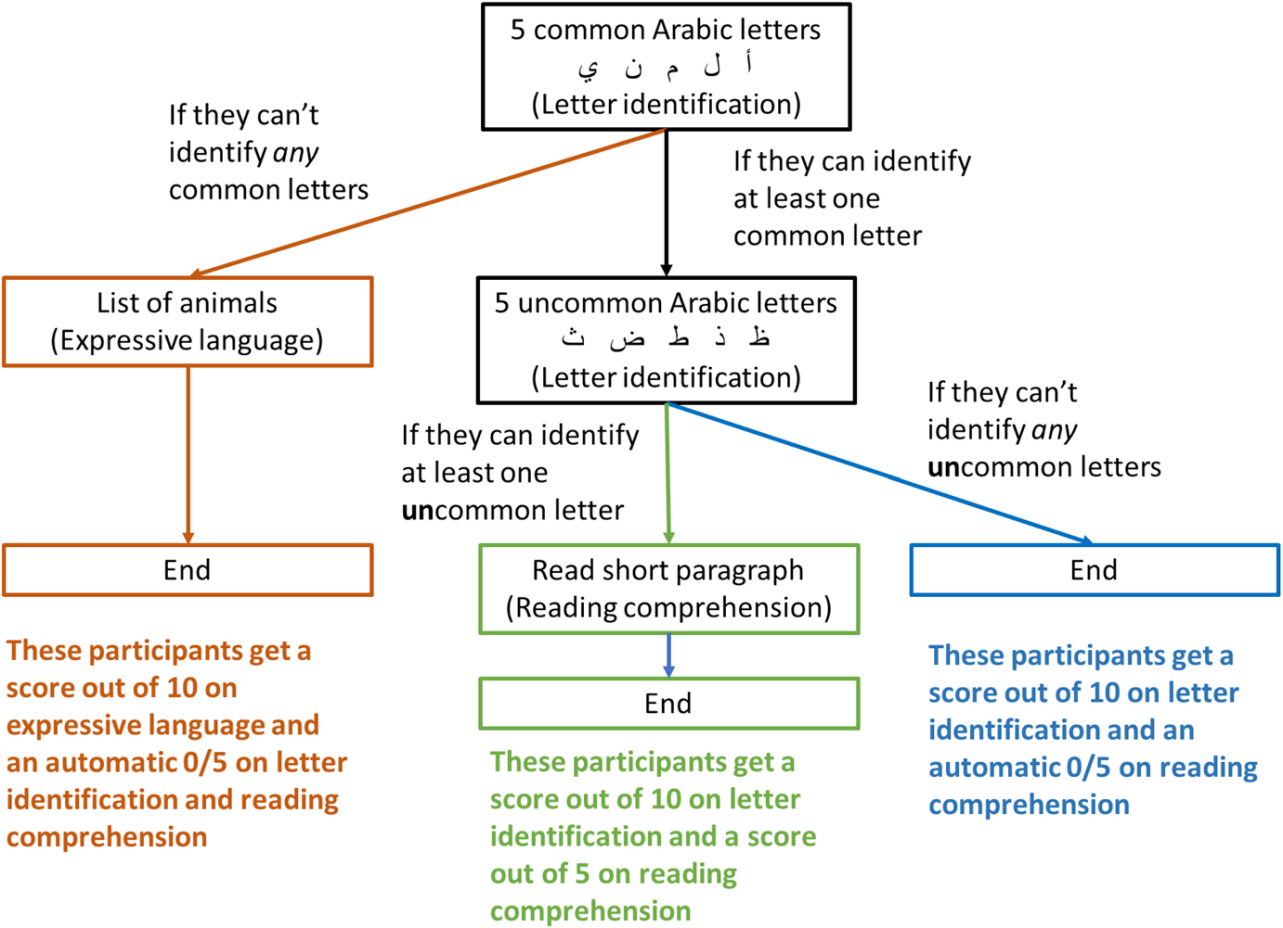
HALDO literacy measure

Children completed a direct assessment of their literacy, using the literacy measure from the Holistic Assessment of Learning and Development Outcomes (HALDO; D’Sa et al., 2019). The HALDO was developed in Uganda to be administered to refugee children aged 4–12 and has been used previously in Lebanon (e.g., Krupar, 2019). We adapted this Arabic-language version used in Lebanon. It consists of three components: letter identification, expressive language, and reading comprehension. It is adaptive, with children *first* completing common letter identification, followed by expressive language if they cannot identify any common letters and uncommon letter identification if they can identify any common letters, and finally by reading a short paragraph if they are able to identify any uncommon letters. Scores can range from 0 to 15, with higher scores indicating higher levels of literacy. See Fig. 1 for how participants move through the task and for their scoring. To ensure consistency of coding among the fieldworkers, two fieldworkers coded the HALDO responses for the first participant at least every second day.

### Statistical Analysis

All analyses presented in this manuscript were pre-registered through OSF (see https://osf.io/7sdh3 and https://osf.io/ztkjd). First, we calculated descriptives and frequencies for all variables, and the correlations between child attitudes toward reading and literacy. We also calculated the inter-rater reliability of the HALDO.

We then ran a series of analyses to understand the relative strength of the association between individual-, household-, and school/community-level factors that may be associated with children’s literacy levels and love of reading. This entailed a series of 4 linear regressions, with literacy and the three child attitudes toward reading measures (PPCATR, YRMQ, and RSCS) as the outcome variables. The predictor variables were child age, child sex, mother age, mother educational level, mother literacy, whether the child had seen someone reading at home, poverty, if the child was enrolled in school, and if there was a COVID-related curfew in place at the time of data collection. Because all mother–child dyads spoke Arabic as children’s first language, we did not include first language as a predictor in the analyses. Following this, we examined the extent to which mode and frequency of school attendance were associated with literacy and attitudes toward reading. We limited the sample to only those children who were currently enrolled in school and then re-ran the same regressions after removing school enrollment and adding mode and frequency of school attendance as predictors. Finally, to understand sex effects, we assessed whether sex of the child moderated the relationship between any significant predictors from the previous analysis and our outcomes, re-running the regressions with sex as a moderator of all significant predictors (e.g., examining interaction effects).

## Results

While some families were more resourced than others, the sample as a whole was challenged at multiple levels. 17.4% of the mothers did not know how to read, and only 56.0% of the children indicating seeing anyone in their family read in the past week. The families had few resources, with an average of 7.92 (*SD* = 1.89) items from the relative wealth checklist. Of note is that while most households had a smartphone (98.1%) and a tv (91.2%), only 7.3% had a computer, and many were missing necessities for the climate in Jordan such as a fan, heater, or refrigerator (with 21.5%, 7.9%, and 13.2% of households, respectively, not having those items).

Most (64.9%) of the children were enrolled in school; this increased to 88.7% when the sample was restricted to just those aged 6 and up, which is the age where school attendance is officially mandatory in Jordan. Few (10.9%) of the mothers were employed. Descriptives for predictors are included in Table 1.

**Table 1:** Descriptives for study variables

	M (or %)	SD	Range
Child age	6.32	1.18	3.5 – 9.17
Child % female	50.0		
Child enrolled in school (%)	64.9		
Mother age	32.61	7.02	20–55
*Mother education (%)*			
No school	10.0		
Grade 1 − 6	27.2		
Grade 7 − 12	53.1		
University	9.7		
Mother can read (%)	82.6		
Relative wealth	7.92	1.89	3–12
Child saw someone read (%)^a^	56.0		
COVID curfew in place (%)	57.5		
Frequency of school attendance	3.64	1.50	1 – 5
*Mode of school attendance (%)*			
In person	9.6		
Online	77.5		
Hybrid (in-person and online)	12.9		

^a^Child reported seeing someone reading at home in the past week

### Levels of Literacy

As shown in Table 2, children’s literacy levels were relatively poor. Given the wide age range of the sample, some of the children were young and would not yet be expected to be able to read. This is shown in Table 2, where very few of the younger children were able to identify any letters. However, those children also tended to have low expressive language skills—being able to list only a few animals.

**Table 2:** Child literacy, as assessed by the HALDO literacy measure

Years old	Unable to identify even one common letter	Unable to identify even one uncommon letter^a^	Expressive language^b^	Unable to read any words in the paragraph^c^	Comprehension questions correctly answered about the paragraph^c^	Total HALDO literacy score^d^
	%	%	M (SD)	%	M (SD)	M (SD)
4	78.4	94.6	3.52 (2.47)	97.4	0.00 (0.00)	3.19 (2.43)
5	61.6	89.0	4.38 (3.58)	91.8	0.00 (0.00)	3.67 (3.38)
6	25.2	53.2	6.00 (2.94)	68.5	0.00 (0.00)	5.27 (3.06)
7	3.8	32.1	5.00 (7.07)	50.9	3.86 (1.46)	6.55 (4.04)
8	5.9	5.9	−	14.3	4.00 (0.95)	9.21 (4.13)

^a^Children who were unable to identify any of the common letters were not asked to identify the uncommon letters here and have been coded as not being able to identify an uncommon letter. ^b^This was only asked of children who were unable to identify even any of the common letters. The max score here is 10, indicating greater levels of expressive language. ^c^Only children who were able to identify at least one common or uncommon letter were asked to read the paragraph and answer questions about it. Note that this is a completely separate group from those who were asked the expressive language questions, since in order to be in that group, a child could not have identified any common letters whereas to be in this group they must have identified at least one common and uncommon letter. ^d^The max score for the HALDO total score is 15, indicating the ability to read a short paragraph and understand its contents

Further, many older children also had limited literacy. Less than 50% of 6-year olds were able to identify even one uncommon Arabic letter, despite this being school-going age (Table 2). Even at age 7, almost a third of participating children were unable to identify any uncommon Arabic letters. Only 38.1% of children progressed to the stage of the literacy test where they were presented with a Grade Two-level paragraph to read. Of those that did progress to this stage, 20.5% could not read any words from the paragraph at all, and on average they were able to read 3.59 words (*SD* = 3.77). Those who were presented with the paragraph to read and were able to read a sufficient proportion were then asked comprehension questions; only 15.6% of children reached this stage, but of those that did, they generally comprehended the paragraph (*M* = 3.95 questions correct, of a possible 5, *SD* = 1.13).

### Relationship Between Child Attitudes Toward Reading and Literacy

As shown in Table 3, the two child-reported measures of attitudes toward reading were moderately correlated with each other. The RSCS was correlated with the mother report of the child attitude toward reading, whereas the YRMQ was not. Both child-reported measures of attitudes toward reading were weakly correlated with literacy; the mother-report was not.

**Table 3:** Pearson correlations between different measures of child attitudes toward reading and child literacy

	RSCS	YRMQ	PPCATR	HALDO
YRMQ	0.587**	−		
PPCATR	0.116*	0.066	−	
HALDO (literacy)	0.118*	0.170*	0.070	−
*M*	7.08	21.73	18.15	5.31
*SD*	1.58	4.93	5.84	3.80
*n*	311	314	319	315

*RSCS*  Reader self-concept scale, *YRMQ*  young reader motivation questionnaire, *PPCATR*  Parents’ perceived attitude toward child reading. *HALDO*  Holistic Assessment of learning and development outcomes. The RSCS, YRMQ, and PPCATR assess child attitudes toward reading, and the HALDO assesses child literacy

**p < 0.001; *p <0 .05

### Predictors of Attitudes Toward Reading

We then ran 6 regression analyses examining the extent to which our predictor variables were associated with attitudes toward reading; the first set of 3 analyses included the full sample, and the second set of 3 analyses was limited to just those children enrolled in school and included the additional variables of frequency and mode of school attendance. The pattern of results when the sample is limited to just those children enrolled in school is broadly in line with when the full sample is included and so we present the second set of analyses here (although see Supplemental Table 1 for the results for the full sample).

The overall models for the YRMQ, RSCS, and PPCATR were all significant (*p* < 0.05). There were different patterns of results depending on the measure used, with only one significant predictor across two measures, and no variable that predicted responses to all three measures of child attitudes to reading (Table 4). Being more impoverished and attending school solely online—as opposed to a hybrid online/in-person model—were associated with higher YRMQ scores. Only seeing someone reading at home in the past week was associated with higher RSCS scores. Being female, seeing someone reading at home, and attending school more frequently were associated with higher PPCATR scores.

**Table 4:** Linear regressions predicting child attitudes toward reading among children enrolled in school (n = 209)

	Model 1, Outcome: YRMQ					Model 2, Outcome: RSCS						Model 3, Outcome: PPCATR				
	*B*	*β*	*SE*	*p*	95% CI	*B*	*β*	*SE*	*p*	95% CI	*B*		*β*	*SE*	*p*	95% CI
*Child age*	−0.12	−0.26	0.36	.727	−0.82, 0.57	0.11	0.07	0.12	.349	−0.12, 0.34	−0.08		−0.01	0.38	.842	−0.83, 0.67
*Child sex*^a^	0.47	0.05	0.98	.111	−0.91, 1.85	−0.02	−0.01	0.23	.939	−0.47, 0.43	**3.66**		**0.34**	**0.76**	** < .001**	**2.17, 5.16**
*Mother age*	−0.04	−0.06	0.71	.502	−1.92, 3.15	−0.03	−0.06	0.02	.072	−0.07, <0.01	−0.09		−0.12	0.06	.115	−0.20, 0.02
*Mother education*^b^																
No school	1.72	0.10	1.96	.380	−2.12, 5.56	0.41	0.07	0.65	.522	−0.85, 1.68	−2.96		−0.15	2.13	.165	−7.12, 1.21
Grade 1–6	0.21	0.02	1.15	.852	−2.03, 2.46	0.06	0.02	0.37	.870	−0.67, 0.79	−1.82		−0.16	1.24	.142	−4.24, 0.61
Grade 7–12	−0.33	−0.04	1.04	.747	−2.34, 1.70	−0.26	−0.08	0.34	.447	−0.92, 0.41	−2.18		−0.20	1.12	.051	−4.37, 0.01
*Mother literacy*	0.61	0.05	1.29	.635	−1.92, 3.15	−0.08	−0.02	0.43	.862	−0.92, 0.80	−0.40		−0.02	1.43	.782	−3.20, 2.41
*Relative wealth*	**−0.67**	**−0.25**	**0.21**	**.001**	**−1.08, −0.26**	−0.06	−0.08	0.07	.322	−0.20, 0.07	−0.20		−0.06	0.23	.385	−0.64, 0.25
*Reading at hom*e^c^	1.25	0.13	0.71	.078	−0.14, 2.64	**0.55**	**0.18**	**0.23**	**.018**	**0.09, 1.00**	**1.59**		**0.15**	**0.76**	**.036**	**0.10, 3.09**
*COVID curfew*	1.57	0.16	0.98	.111	−0.36, 3.49	0.46	0.15	0.32	.153	−0.17, 1.09	−0.58		−0.05	1.06	.587	−2.65, 1.50
*Frequency of attendance*	0.43	0.14	0.25	.080	−0.05, 0.91	0.14	0.14	0.08	.073	−0.01, 0.30	**0.61**		**0.17**	**0.27**	**.022**	**0.09, 1.13**
*Mode of attendance*^*d*^																
In person	−0.77	−0.29	1.44	.593	−3.59, 2.05	−0.15	0.03	0.47	.756	−1.07, 0.78	−1.47		−0.09	1.53	.339	−4.48, 1.54
Online	**3.07**	**−0.05**	**1.22**	**.011**	**−5.49, −0.70**	−0.61	−0.18	0.39	.120	−1.38, 0.16	1.46		0.12	1.30	.261	−1.09, 4.02
R^2^	0.119					0.094					0.217					

Bolded variables are significantly predictive in that model. YRMQ = Young Reading Motivation Questionnaire, RSCS = Reader Self-Concept Scale, PPCATR = parents’ perception of their child’s attitude toward reading. ^a^For child sex, 0 is male, and 1 is female. ^b^For maternal highest level of education, having gone to university is the reference level. ^c^This refers to whether the child has seen someone reading at home in the past week. ^d^Hybrid (both in-person and online) attendance is the reference level. Frequency and mode of attendance refer to school attendance

### Predictors of Literacy

We then conducted a regression to see the extent to which our predictor variables were associated with child literacy. As above, we first ran this analysis in the whole sample and then again in just those enrolled in school. As the pattern of results differed substantially, we present both analyses in Table 5.

**Table 5:** Regressions predicting child literacy

	Model 1, Full sample					Model 2, Those enrolled in school					
	*B*	*β*	*SE*	*p*	95% CI	*B*		*β*	*SE*	*p*	95% CI
*Child age*	**1.45**	**0.45**	**0.22**	** < .001**	**1.01, 1.88**	**1.53**		**0.41**	**0.25**	** < .001**	**1.04, 2.02**
*Child sex*^a^	−0.24	−0.03	0.38	.538	−0.98, 0.51	0.01		< .001	0.50	.990	−0.97, 0.99
*Enrolled in school*	0.50	0.06	0.52	.338	−0.52, 1.51						
*Mother age*	−0.04	0.08	0.03	.129	−0.10, 0.01	−0.01		−0.02	0.04	.770	−0.08, 0.06
*Mother education*^b^											
No school	−1.40	−0.11	1.14	.218	−3.63, 0.83	−1.62		−0.11	1.41	.251	−4.38, 1.15
Grade 1–6	**−2.56**	**−0.31**	**0.72**	** < .001**	**−3.96, −1.15**	**−1.90**		**−0.24**	**0.81**	**.019**	**−3.49, −0.31**
Grade 7–12	**−1.80**	**−0.24**	**0.65**	**.006**	**−3.07, −0.52**	**−1.88**		**−0.26**	**0.73**	**.010**	**−3.32, −0.45**
*Mother literacy*	1.33	0.14	0.76	.077	−0.14, 2.81	**1.92**		**0.20**	**0.92**	**.036**	**0.12, 3.71**
*Relative wealth*	0.19	0.09	1.07	.079	−0.02, 0.40	0.09		0.04	0.15	.526	−0.20, 0.38
*Reading at home*^c^	−0.27	−0.04	0.38	.483	−1.02, 0.48	−0.09		−0.01	0.50	.863	−1.07, 0.90
*COVID curfew*	−0.16	−0.02	0.39	.677	−0.93, 0.60	0.39		0.05	0.70	.573	−0.97, 1.76
*Frequency of attendance*						0.20		0.08	0.18	.244	−0.14, 0.55
*Mode of attendance*^d^											
In person						**−2.61**		**−0.22**	**1.01**	**.009**	**−4.58, −0.64**
Online						**−1.76**		**−0.21**	**0.85**	**.039**	**−3.44, −0.09**
*R*^2^		0.279					0.275				
*n*		291					178				

Frequency and mode of attendance in school were not included as predictors in Model 1 which included the full sample, as this would have limited the sample to those who were enrolled in school. Being enrolled in school was not included as a predictor in Model 2 as the sample for Model 2 was limited to those who were enrolled in school. Frequency and mode of attendance refer to school attendance. Bolded variables are significantly predictive in that model. ^a^ For child sex, 0 is male and 1 is female. ^b^ For maternal highest level of education, having gone to university is the reference level. ^c^ This refers to whether the child has seen someone reading at home in the past week. ^d^ Hybrid (both in person and online) attendance is the reference level. Frequency and mode of attendance refer to school attendance

In both models—and as would be expected—older children had higher literacy scores. For every year increase in age, children scored an additional 1.5 points on the HALDO. In both models, maternal education was associated with child literacy, with the children of mothers whose highest level of education was either between Grade 1–6 or between Grade 7–12 having lower literacy than those children whose mothers had attended university. In the sample of only those enrolled in school, when mothers were literate, children had higher literacy scores. While frequency of attendance at school was not predictive of literacy, mode of attendance was: children attending school either in-person or online had lower literacy than those attending school both in-person and online (hybrid). With the exception of age, each of these predictors was a similarly strong predictor of child literacy (see Table 5, *β* values).

### Moderation by Sex

Finally, we ran the regressions presented in Tables 3 and 4, but tested for a moderation (interaction effect) of each significant predictor by child sex. There was no moderation of household wealth or mode of school attendance for the YRMQ, nor moderation for seeing someone reading at home or for frequency of school attendance for the PPCATR (*p*s > 0.05). There was a significant child sex X seeing someone reading at home interaction for the RSCS (*B* = 1.23, *SE* = 0.45, *p* = 0.006, 95% CI: 0.36, 2.10): boys’ attitudes toward reading were consistent regardless of whether they had seen someone reading at home, whereas girls who had not seen anyone reading at home had worse attitudes toward reading and those who seen someone reading at home had better attitudes toward reading. For literacy, there were no moderation of child age, maternal education, or relative wealth for those not in school, nor were there moderations for child age, maternal education, maternal literacy, or mode of school attendance for those who were in school (*p*s > 0.05).

## Discussion

Refugee children in Jordan face multiple challenges to their educational access and attainment. In this study, we sought to understand levels of literacy among a sample of Syrian refugee children in Jordan and to assess how multilevel (child-, mother-, household-, and school-level) challenges were associated with literacy and attitudes toward reading in early childhood. There are three main findings from this study: 1) rates of literacy among the children were low; 2) child-, mother-, household-, and school-level variables were associated with children’s differences in literacy; and 3) there is a need to validate mother- and child-reported Arabic-language child attitude toward reading measures.

Children had relatively low levels of literacy in this sample. For English-speaking children, we may expect a 4-year-old to be able to identify some letters, a 5–6-year-old to be able to write their name, and a 6–7-year-old to recognize ¬100 words (Horowitz-Kraus et al., 2017). Due to its diglossic nature and the many different dialects, it may be more challenging for children to learn to read Arabic (Eghbaria-Ghanamah et al., 2020; Khamis-Dakwar et al., 2022; Schiff et al., 2018). While there is less information on what would be normative for Arabic-speaking youth, research suggests that Arabic-speaking 5–6-year olds tend to be able to write and read some letters, and those aged 6–7 tend to be able to read multiple words (Aram et al., 2013; Hassunah‑Arafat, Aram, & Korat, 2021; Khoury-Metanis & Khateb, 2022). In our sample, by contrast, 39% of 5-year olds could not identify even one common letter and more than 50% of 6-year olds could not identify even one uncommon letter. Most children were not able to read any words, and their expressive language was generally not strong. Some of this is explained by age—with the older children generally scoring higher on the HALDO and displaying different types of literacy skills—but even the older children who were enrolled in school did not tend to be able to read more than one or two words. This is particularly troubling given that early reading skill development in Jordan consists of learning to hold a pencil, the alphabets, letter sounds, and how the shape of letters changes depending on where it is in a word, and thus, these children should have had some exposure to the types of literacy activities assessed by the HALDO.

This leads to the second study finding, that a variety of multilevel (child-, mother-, household-, and school-level) factors predicted literacy in this sample. Refugee children in general face multiple challenges to their literacy and education (UNHCR, 2016). Some of our findings are in line with expectations: socioeconomic status, maternal education, and maternal literacy have been robustly associated with academic achievement and attainment, including literacy (e.g., Harding et al., 2015; Hassunah‑Arafat et al., 2021; Skibbe et al., 2008; Wamba, 2010). More surprising was that hybrid teaching was associated with higher levels of literacy than either solely in-person or solely online teaching. While there is good evidence for the efficacy of blended learning (e.g., Means et al., 2013), few studies have examined its use in early childhood, particularly among refugee children living in low- or middle-income countries. One possibility is that the type of schools which offered hybrid teaching differed in positive ways from solely in-person or solely online schools, and thus, it was not the nature of delivery itself but rather some pre-existing differences between the schools and associated environments of the children attending those schools. Although we did not find an impact for school closures at the time of data collection in predicting literacy, COVID has led to reduced opportunities for learning to read for these refugee children in general, with schools in Jordan intermittently closed across 2020 and 2021 (FPA, 2021). Although the Jordanian government made a variety of efforts to minimize learning disruptions (Holleis, 2021)—including putting some educational content on tv and providing some computers—much of this would not have been accessible to our study sample, where children were unlikely to have regular access to a smart phone, much less a computer. Given government closures of in-person schooling in response to COVID-19, another possibility for our hybrid learning finding is that those schools which were in-person only would have been completely closed, those which conducted classes online only may have had too many barriers to attendance for the children in this study, whereas those with blended learning options may have enabled to broadest levels of attendance. In general, our results support the important role of both family- and school-level influences in the development of child literacy skills (Dong et al., 2020; Isik-Ercan et al., 2017; Kim et al., 2020; Pacheco & Mata, 2022; Taylor et al., 2000).

A lack of conceptual clarity surrounds the assessment of children’s attitudes toward reading (Davis et al., 2018), and there are no validated measures to assess attitudes toward reading among Arabic-speaking children. We used here one mother-report measure (PPCATR) and two child-report measures (RSCS, YRMQ), all of which had acceptable reliability in this sample, but their convergent and predictive validity was inconsistent. The two child-reported measures were strongly correlated with each other but were inconsistently associated with child literacy, and the mother-reported measure correlated weakly with one of the child-report measures (RSCS) but not with the other (YRMQ) or with child literacy. Our research highlights the need to validate Arabic language attitude toward reading measures, so that this construct can be meaningfully assessed in region.

## Strengths and Limitations

This study has a number of strengths, including a large sample of refugee children and their mothers, the use of self-reports and direct assessments, and the inclusion of both child and mother reports. It has three main limitations. First, the sample was drawn from community-based organizations and is not necessarily representative of the wider Syrian refugee population in Jordan. We cannot use this data, for instance, to provide nationally representative estimates of levels of literacy among Syrian refugee children in Jordan. Second, the data are cross-sectional, and so we can only assess associations between our variables. Although some of the associations appear directional, we cannot infer causal explanations. Third, we do not have information on expected literacy milestones by age in Jordan, nor age-normed literacy levels for refugee and non-refugee children in Jordan using the HALDO, and so it is not possible to compare our findings to country- or population-specific norms.

## Conclusion

This is one of the first studies of levels and predictors of literacy among Syrian refugee children, and indeed of refugee kids generally. It is critical to collect this literacy data so that policymakers and practitioners are able to intervene in those populations and with those children where it is most needed. We found that there were very low rates of literacy among Syrian refugee children in Jordan, suggesting an urgent need for effective intervention.

Understanding how child-, parent-, household-, and school-level factors predict children’s literacy and attitudes toward reading and their reading abilities is crucial for practitioners, researchers, and policymakers in this region, so that they effective interventions can be designed and implemented. That maternal education, maternal literacy, and mode of school attendance were predictive of child literacy suggests potential targets for intervention in this vulnerable population. Future research should examine the efficacy of family- and school-level programs at addressing this literacy deficit.

Literacy levels in early childhood are associated with a variety of outcomes across the lifespan. They serve as the bedrock for further learning (e.g., Kennedy et al., 2012). For refugee children, developing good literacy skills may be particularly important to overcome the many challenges and disadvantages that they face.

## Supplementary Information

Below is the link to the electronic supplementary material.

Supplementary file1 (DOCX 19 kb)
