# Supplementary material for: Predictors of Literacy and Attitudes Toward Reading Among Syrian Refugee Children in Jordan
Source: Int J Early Child. 2022 Sep 9:1–21. Online ahead of print. doi: 10.1007/s13158-022-00334-x (PMC9461418; doi:10.1007/s13158-022-00334-x)
Supplement: Supplementary file 1 — Supplementary file1 (DOCX 19 kb) [file 13158_2022_334_MOESM1_ESM.docx]

*Supplemental Table 1*. Linear regressions predicting child attitudes toward reading among all children in the study (*n* = 322)

|  | **Model 1, Outcome: YRMQ** | | | | | **Model 2, Outcome: RSCS** | | | | |  | **Model 3, Outcome: PPCATR** | | | | |
| --- | --- | --- | --- | --- | --- | --- | --- | --- | --- | --- | --- | --- | --- | --- | --- | --- |
|  | *B* | *β* | *SE* | *p* | 95% CI | *B* | *β* | *SE* | *p* | 95% CI | *B* | | *β* | *SE* | *p* | 95% CI |
| Child age | -0.26 | -0.06 | 0.34 | .440 | -0.91, 0.39 | 0.05 | 0.04 | 0.11 | .625 | -0.15, 0.26 | 0.01 | | 0.002 | 0.38 | .982 | -0.71, 0.73 |
| Child sex^a^ | 0.46 | 0.05 | 0.59 | .431 | -0.66, 1.59 | 0.21 | 0.07 | 0.19 | .261 | -0.15, 0.57 | **3.68** | | **0.32** | **0.65** | **<.001** | **2.43, 4.94** |
| Mother age | -0.05 | -0.07 | 0.04 | .263 | -0.13, 0.04 | -0.02 | -0.07 | 0.01 | .254 | -0.04, 0.01 | -0.03 | | -0.03 | 0.05 | .576 | -0.12, 0.07 |
| Mother education^b^ |  |  |  |  |  |  |  |  |  |  |  | |  |  |  |  |
| No school | -0.38 | -0.02 | 1.73 | .827 | -3.71, 2.95 | 0.14 | 0.03 | 0.56 | .801 | -0.93, 1.22 | -3.43 | | -0.18 | 1.95 | .079 | -7.16, 0.31 |
| Grade 1-6 | -0.80 | -0.07 | 1.10 | .472 | -2.92, 1.32 | -0.17 | -0.05 | 0.35 | .628 | -0.85, 0.51 | **-2.61** | | **-0.20** | **1.23** | **.035** | **-4.97, -0.25** |
| Grade 7-12 | -0.50 | -0.05 | 1.00 | .618 | -2.43, 1.43 | -0.23 | -0.07 | 0.32 | .476 | -0.84, 0.39 | **-3.02** | | **-0.26** | **1.12** | **.007** | **-5.15, -0.87** |
| Mother literacy | 0.07 | 0.01 | 1.16 | .956 | -2.16, 2.29 | -0.04 | -0.01 | 0.37 | .909 | -0.76, 0.68 | 0.41 | | 0.03 | 1.31 | .758 | -2.12, 2.93 |
| Relative wealth | **-0.44** | **-0.17** | **0.16** | **.008** | **-0.75, -0.13** | -0.05 | -0.06 | 0.05 | .363 | 0.05, 0.87 | -0.06 | | -0.02 | 0.18 | .762 | -0.41, 0.30 |
| Reading at home^c^ | 0.81 | 0.08 | 0.59 | .170 | -0.32, 1.94 | **0.50** | **-0.16** | **0.19** | **.008** | **0.14, 0.87** | 0.28 | | 0.03 | 0.66 | .665 | -0.98, 1.54 |
| Child enrolled in school | 0.53 | 0.05 | 0.80 | .506 | -1.00, 2.06 | **-0.22** | -0.07 | 0.25 | .387 | -0.71, 0.27 | -0.01 | | -0.001 | 0.89 | .989 | -1.72, 1.70 |
| COVID curfew | -1.07 | -0.11 | 0.60 | .075 | -2.22, 0.08 | -0.21 | -0.07 | 0.19 | .268 | -0.58, 0.16 | 0.85 | | 0.07 | 0.67 | .205 | -0.43, 2.13 |
| R^2^ | .213 | | | | | .206 | | | | | .361 | | | | | |

YRMQ = Young Reading Motivation Questionnaire, RSCS = Reader Self Concept Scale, PPCATR = parents’ perception of their child’s attitude towards reading. ^a^ For child sex, 0 is male and 1 is female. ^b^ For maternal highest level of education, having gone to university is the reference level. ^c^ This refers to whether the child has seen someone reading at home in the past week. Bolded variables are significantly predictive in that model.
